# Cross-Ancestry Comparison of Aptamer and Antibody Proteomics Measures

**DOI:** 10.21203/rs.3.rs-5968391/v1

**Published:** 2025-02-13

**Authors:** Jayna C. Nicholas, Daniel H. Katz, Usman A. Tahir, Catherine L. Debban, Francois Aguet, Thomas Blackwell, Russell P. Bowler, K. Alaine Broadaway, Jingsha Chen, Clary B. Clish, Josef Coresh, Elaine Cornell, Daniel E. Cruz, Rajat Deo, Margaret F. Doyle, Peter Durda, Lynette Ekunwe, James S. Floyd, Dipender Gill, Xiuqing Guo, Ron C. Hoogeveen, Craig Johnson, Leslie A. Lange, Yun Li, Alisa Manning, James B. Meigs, Michael Y. Mi, Josyf C. Mychaleckyj, Nels C. Olson, Katherine A. Pratte, Brucy M. Psaty, Alexander P. Reiner, Peifeng Ruan, Magdalena Sevilla-Gonzalez, Amil M. Shah, Quan Sun, Russell P. Tracy, Jia Wen, Alexis C. Wood, James G. Wilson, Kristin L. Young, Bing Yu, Mary R. Rooney, Ani Manichaikul, Ruth Dubin, Karen L. Mohlke, Stephen S. Rich, Jerome I. Rotter, Peter Ganz, Robert E. Gerszten, Kent D. Taylor, Laura M. Raffield

**Affiliations:** 1 Department of Genetics, University of North Carolina at Chapel Hill, Chapel Hill, NC, USA,; 2 Cardiovascular Medicine, Stanford University, Stanford, CA, USA; 3 Division of Cardiovascular Medicine, Beth Israel Deaconess Medical Center, Boston, MA, USA; 4 Department of Genome Sciences, University of Virginia, Charlottesville, VA, USA; 5 Broad Institute, Cambridge, MA, USA; 6 University of Michigan, Ann Arbor, MI, USA; 7 National Jewish Health, National Jewish Health, Denver, CO, USA; 8 Johns Hopkins Bloomberg School of Public Health, Baltimore, MD, USA; 9 Metabolomics Platform, Broad Institute, Cambridge, MA, USA; 10 Department of Population Health, Institute for Optimal Aging, New York, NY, USA; 11 Larner College of Medicine at the University of Vermont, Burlington, VT, USA; 12 Division of Cardiovascular Medicine, University of Pennsylvania, Philadelphia, PA, USA; 13 Department of Pathology and Laboratory Medicine, Larner College of Medicine at the University of Vermont, Burlington, VT, USA; 14 University of Mississippi Medical Center, Jackson, MS, USA; 15 School of Medicine, University of Washington, Seattle, WA, USA; 16 Sequoia Genetics, London, London, UK; 17 The Institute for Translational Genomics and Population Sciences, Department of Pediatrics, The Lundquist Institute for Biomedical Innovation at Harbor-UCLA Medical Center, Torrance, CA, USA; 18 Medicine, Cardiovascular Research, Baylor College of Medicine, Houston, TX, USA; 19 University of Washington, Seattle, WA, USA; 20 School of Medicine, Colorado Anschutz Medical Campus, Aurora, CO, USA; 21 Department of Biostatistics, University of North Carolina at Chapel Hill, Chapel Hill, NC, USA; 22 Broad Institute, Harvard University, Massachusetts General Hospital, Boston, MA, USA; 23 Department of Medicine, Division of General Internal Medicine, Broad Institute, Boston, MA, USA; 24 Department of Medicine, Division of Cardiovascular Medicine, Beth Israel Deaconess Medical Center, Boston, MA, USA; 25 Department of Biostatistics, National Jewish Health, Denver, CO, USA; 26 Cardiovascular Health Research Unit, Departments of Medicine and Epidemiology, University of Washington, Seattle, WA, USA; 27 Fred Hutchinson Cancer Research Center, University of Washington, Seattle, WA, USA; 28 UT Southwestern, Dallas, TX, USA; 29 Clinical and Translational Epidemiology Unit, Mongan Institute, Massachusetts General Hospital, Boston, MA, USA; 30 Department of Medicine, Harvard Medical School, Boston, MA, USA; 31 Programs in Metabolism and Medical & Population Genetics, Broad Institute, Harvard University, Massachusetts General Hospital, Cambridge, MA, USA; 32 Harvard Medical School, Boston, MA, USA; 33 USDA/ARS Children’s Nutrition Research Center, Department of Pediatrics, Baylor College of Medicine, Houston, TX, USA; 34 Deparment of Cardiology, Beth Israel Deaconess Medical Center, Boston, MA, USA; 35 Department of Epidemiology, University of North Carolina at Chapel Hill, Chapel Hill, NC, USA; 36 UT Health, School of Public Health, Houston, TX, USA; 37 Department of Epidemiology, Johns Hopkins Bloomberg School of Public Health, Baltimore, MD, USA; 38 Division of Cardiology, Department of Medicine, University of California, San Francisco, San Francisco, CA, USA

## Abstract

Measures from affinity-proteomics platforms often correlate poorly, challenging interpretation of protein associations with genetic variants (pQTL) and phenotypes. Here, we examined 2,157 proteins measured on both SomaScan 7k and Olink Explore 3072 across 1,930 participants with genetic similarity to European, African, East Asian, and Admixed American ancestry references. Inter-platform correlation coefficients for these 2,157 proteins followed a bimodal distribution (median r=0.30). Protein measures from each platform were associated with genetic variants (pQTLs), and one-third of the pQTL signals were driven by protein-altering variants (PAVs). We highlight 80 proteins that correlate differently across ancestry groups likely due to differing PAV frequencies by ancestry. Furthermore, adjustment for PAVs with opposite directions of effect by platform improved inter-platform protein measure correlation and resulted in more concordant genetic and phenotypic associations. Hence, PAVs need to be accounted for across ancestries to facilitate platform-concordant and accurate protein measurement.

## Main

Genetic and epidemiological studies of circulating protein abundances yield insights that promise to enhance discovery of clinical biomarkers and drug targets. Recent advances in multiplexed affinity-based proteomics platforms have made biobank-scale studies of the circulating proteome possible ^1–6^. Currently, the leading affinity-based proteomics platforms are SomaScan aptamer assays, which use oligonucleotide affinity probes to quantify 1,100–11,000 proteins in a plasma/serum sample ^7^, and Olink proximity extension assays (PEA), which employ conjugated antibody-nucleic acid assay probes to quantify 48–5,400 proteins ^8^. With efficient pipelines for sample analysis, and lower intra-assay variability compared to many existing mass-spectrometry (MS) proteomics approaches ^9,10^, both platforms hold great potential for continued use in biobank-scale studies and future clinical application.

However, existing comparisons of SomaScan and Olink platforms have shown that some protein measures are not concordant (median inter-platform correlation=0.38–0.50) ^11–16^, which challenges the accuracy of protein associations observed, especially as there is no “gold-standard” reference measure for most proteins. Additionally, there is no reported method to reconcile protein measure discordance across platforms, preventing effective cross-platform meta-analysis of many protein measures. Furthermore, existing comparisons of SomaScan and Olink protein measures are limited to populations of predominantly one ancestry; it remains unknown if assay performance varies across diverse ancestries.

Genetic variants may alter circulating protein abundances through many biological mechanisms, including alterations to transcriptional or translational efficiencies and post-translational modifications (PTMs) that impact downstream protein stabilities. Genetic variation may also impact protein structure or post-translational modifications (PTMs) in a manner that alters epitope binding to affinity reagents. Protein quantitative trait loci (pQTL), or associations between circulating protein abundances and genetic variants either within or nearby (*cis-*) to the protein encoding-gene or elsewhere in the genome (*trans-*), are commonly detected in large-scale studies.^1–6^. The detection of a *cis*-pQTL for a given protein measure is often used as a metric to confirm on-target affinity-probe binding^12,15,16^; existing studies comparing SomaScan and Olink measures have established that the detection of a *cis*-pQTL is a strong predictor of inter-platform correlation coefficients for a given protein ^12,15,16^.

Nevertheless, interpreting *cis-* and *trans*-pQTL remains complicated, as protein altering variants (PAVs) may result in pQTL associations that reflect differential epitope binding to alternate proteoforms ^12,17–19^ rather than differential protein abundances. As these genetic variants may vary in frequency across populations, such variants could drive systematic differences in protein measures ^20^. Failure to detect and account for such genetic variation may confound results obtained across ancestries and introduce a source of bias ^21^. This holds important implications for many methods, including Mendelian Randomization (MR), which relies on genetic associations reflecting effects on protein abundances to infer protein genetic liability and drug target efficacy.

Here, we evaluate potential genetic drivers of inter-platform and inter-ancestry variation between SomaScan 7k and Olink Explore 3072 protein measurements from 1,930 participants from the Multi-Ethnic Study of Atherosclerosis (MESA). For the 2,157 proteins measured on both platforms in the same plasma sample, we assess inter-platform correlation and examine the cross-platform concordance of pQTL and epidemiological associations within and across ancestry groups.

## Results

### Participant demographics

The SomaScan 7k and Olink Explore 3072 platforms were used to generate protein abundance measures at MESA exam 1 (2000 – 2002) in EDTA-plasma samples from 1,930 participants. The mean age of this cohort was 58.2 years (+/− 8.8), with mean body mass index (BMI) of 28.1 kg/m^2^ and mean eGFR of 80.7 mL/min/1.73m^2^ (**Table S1**). Most participants exhibited >0.5 genetic similarity to African (AFR, n=439), Admixed American (AMR, n=231), East Asian (EAS, n=277), or European (EUR, n=939) 1000 Genomes Project reference populations, based on global ancestry proportion estimates (**Table S1**), enabling stratification by genetic similarity for some analyses.

### Proteomics platforms

The SomaScan 7k assay in MESA includes 7,289 aptamers, targeting 6,407 distinct human UniProt IDs. The Olink Explore 3072 assay includes 2,941 antibody complexes, targeting 2,923 distinct human UniProt IDs. Additionally, SomaScan offers two versions of data: with adaptive-normalization by maximum likelihood performed on quality control samples only (ANML-QC) or performed on all participant samples (ANML-SMP). Similar numbers of protein principal components (PCs) were required to explain 95% of the variation in protein measures from each platform (940 ANML-SMP SomaScan vs. 880 Olink) (**Figure S1**), despite the different number of proteins measured.

**Figure 1.**
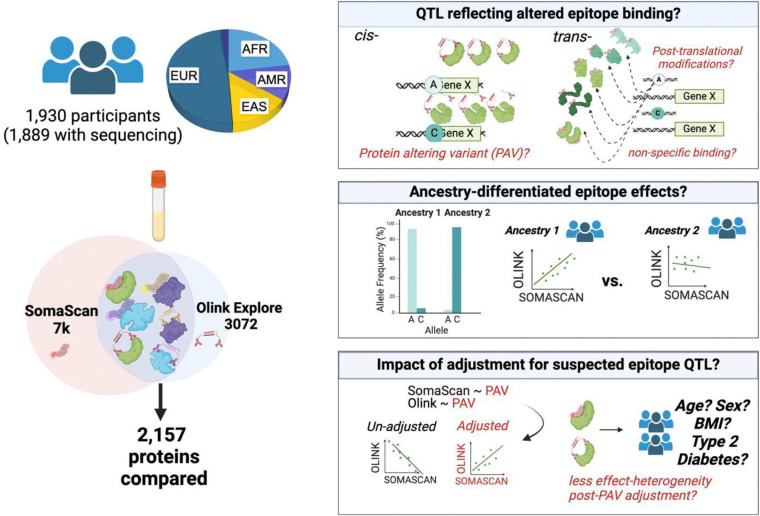
Graphical summary of main analyses with **(Left)** depiction of relative numbers of participants clustering with each ancestry-reference (>0.5 similarity to 1000G ancestry cluster). A small portion of participants (n=44, 2%) did not cluster with any ancestry reference population. 2,157 proteins measured on both SomaScan 7k and Olink Explore 3072 were compared in present analyses. **(Right, top panel)** Examples illustrating how *cis*- and *trans*- pQTL associations may capture differences in epitope binding rather than differences in protein abundance. (**Right, middle panel**) Genetic variants which alter epitope binding efficiency and differ in frequency across ancestries may systematically bias affinity-probe measurements in one or more ancestry groups, resulting in systematic differences in measurements, and subsequently, differences in cross-platform correlation in at least one ancestry. (**Right, bottom panel**) A central hypothesis of this study is that adjusting an individual’s protein measures for variants which likely impact affinity probe binding, rather than abundance, may strengthen accuracy of protein measures on the impacted platform, resulting in stronger inter-platform agreement of protein measures and more concordant downstream epidemiological and genetic associations.

Here, we compared SomaScan 7k and Olink Explore 3072 protein abundances for the 2,157 distinct human proteins that were assessed on both platforms in 1,889 MESA participants of diverse ancestry (Figure 1). Protein measures from each platform were standardized and inverse normal transformed to facilitate comparison of SomaScan Relative Fluorescence Units (RFU) and Olink Normalized Protein Expression (NPX) values. Although both platforms offer multiple affinity-probes for a subset of proteins, we present results corresponding to measures obtained from the SomaScan aptamer and one Olink antibody-conjugate probe pair with the highest inter-platform correlation (unless otherwise stated). Results for all probes are in the supplement.

### Correlation and cross-ancestry differences in SomaScan and Olink measures

**Extended Data Figure 1.**
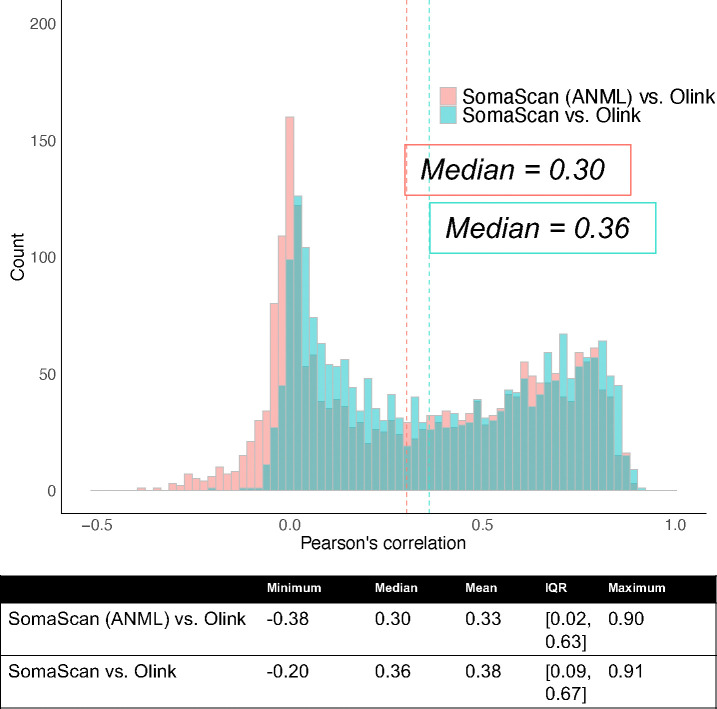
Inter-platform Pearson’s correlation estimates for 2,157 proteins measured with both SomaScan 7k aptamer and Olink Explore 3072 antibodies. Results from the comparison of one probe pair per UniProt ID are depicted in this figure. Correlation coefficients between base-normalized SomaScan and Olink Explore measures are plotted in blue. Correlation coefficients obtained by comparison of SomaScan measures with adaptive-normalization by maximum likelihood (ANML) performed on all samples (ANML-SMP) vs. Olink Explore measures plotted in pink.

To evaluate cross-platform agreement of protein measures, we computed Pearson’s correlation of transformed protein measures obtained from probes targeting the same protein. Consistent with prior studies, correlation coefficients comparing SomaScan ANML-QC and Olink followed a bimodal distribution with a median Pearson’s correlation of 0.36 (Quartile 1=0.09, Quartile 3=0.67), and a median of 0.30 (Quartile 1=0.02, Quartile 3=0.63) comparing SomaScan ANML-SMP measures and Olink (Extended Data Figure 1, **Figure S2, Table S2**). As prior studies demonstrate ANML-SMP SomaScan normalization lowers intra-assay coefficients of variation ^17,22^ and maximizes potential for genetic discovery, and per SomaLogic recommendations, we used ANML-SMP SomaScan values throughout remaining analyses.

**Extended Data Figure 2.**
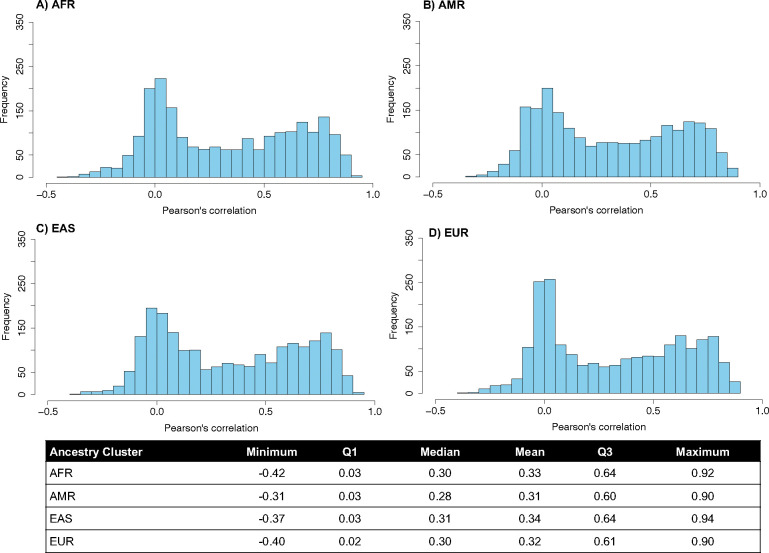
Inter-platform Pearson correlation estimates by ancestry for 2,157 SomaScan SeqIDs and 2,157 Olink IDs targeting the 2,157 UniProt IDs measured on both platforms. Table displays values of the minimum, first quartile (Q1), median, mean, third quartile (Q3), and maximum Pearson’s r value observed within individuals of each ancestry cluster.

Overall, platforms followed similar patterns of agreement within each ancestry, evidenced by similar median correlations and distributions of Pearson’s correlation coefficients observed within each ancestry [median Pearson’s r AFR=0.30, AMR=0.28, EAS=0.31, EUR=0.32] (Extended Data Figure 2, **Table S2**). However, 80 protein targets exhibited significant differences in assay agreement across ancestries (Cochran’s Q P<1.85*10^−5^, Bonferroni adjustment for the total number of probe pairs (2,708)) (**Table 1, Table S2**), suggesting systematic differences in measurement accuracy across ancestries, on one or both platforms.

### Influence of genetic variants on protein levels

To explore genetic drivers of platform-discordance and cross-ancestry heterogeneity in inter-platform correlation estimates, we mapped associations between genetic variants and SomaScan/Olink protein measures in 1,889 MESA participants with whole genome sequencing (WGS) data, excluding first-degree relatives. As *cis*-pQTL are in close proximity to the protein-encoding gene, these associations are most likely to reflect on-target epitope binding, and in a subset of coding variants, genetic variation that impacts epitope binding (Figure 1).

**Figure 2.**
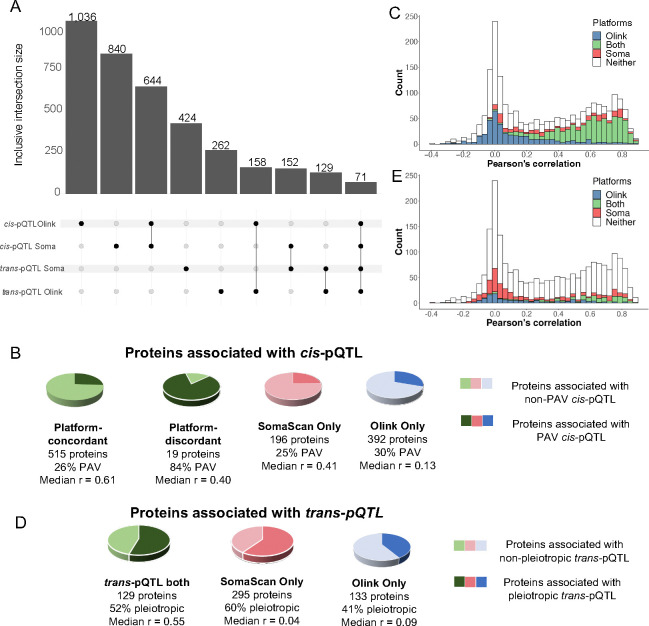
Comparison of genetic signals detected for 2,157 proteins measured on both SomaScan and Olink Explore. (A) UpSet plot depicting total counts of proteins with *cis*- and *trans*-pQTL on each platform. (B) Pie charts depicting the percentage of proteins associated with a platform-concordant, platform-discordant, SomaScan specific, or Olink specific *cis*-pQTL which were also associated with a PAV. (C) Histogram of Pearson’s correlation coefficients (r values), with bars colored to indicate which proteins in bin had a significant *cis*-pQTL detected on SomaScan, Olink, both, or neither platform. (D) Pie charts depicting the percentage of proteins associated with a pleiotropic *trans*-pQTL (signal associated with >=5 protein measures) among the proteins with a *trans-*pQTL on both platforms or one platform only. (E) Histogram of Pearson’s correlation coefficients (r values), with bars according to whether proteins in bin had a significant *trans*-pQTL detected on SomaScan, Olink, both, or neither platform.

Here, we identified significant (see Methods) *cis*-pQTL credible sets for 840 proteins on SomaScan (**Table ST3**) and 1,036 proteins on Olink (**Table 2, ST4**, Figure 2A). 79% and 93% of SomaScan credible sets and 65% and 98% of Olink credible sets reported herein were within 1 Mb regions previously reported in deCODE and UK Biobank, respectively (**Table 2**). A higher proportion of secreted proteins had a significant *cis*-pQTL association compared to intracellular and membrane proteins (**Figure S3**).

**Extended Data Figure 3.**
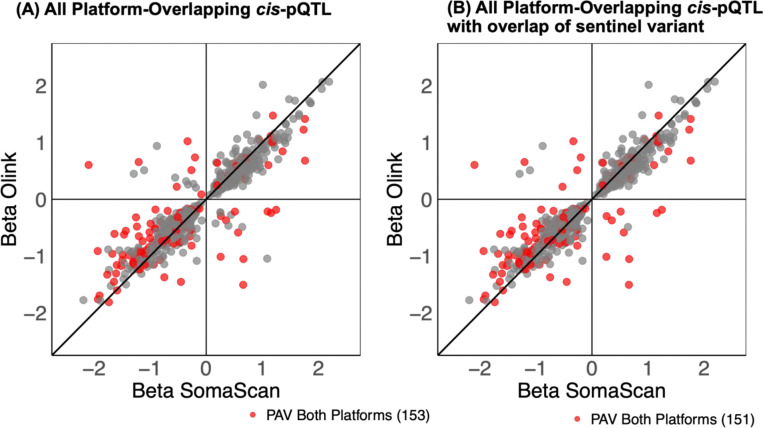
Sentinel variant *cis-*pQTL effect-size comparison for platform-overlapping *cis*-pQTL identified in one-probe-per-UniProt analysis, with points colored according to whether a PAV for the protein-encoding gene was present in one or both platform’s credible set. (A) Effect-size comparison across all platform-overlapping credible sets. (B) Effect-size comparison across platform-overlapping credible sets for which at least one platform’s sentinel variant shared.

As PAVs can impact affinity-probe binding, *cis*-pQTL associations driven by PAVs may reflect assay interference, particularly when the association is detected on only one platform or detected on both with different effect-directions between platforms. Among the 196 and 392 proteins with a *cis*-pQTL association detected with SomaScan or Olink measures only, similar proportions were associated with at least one PAV (25% SomaScan, 30% Olink) (Figure 2B). We further classified overlapping signal pairs by concordance of sentinel variant effect-directions (**Figure S4**). While most of these signals were led by variants with concordant directions of association across platforms (referred to herein as “platform-concordant” signals), we identified 19 proteins with platform-overlapping *cis*-pQTL credible sets for which sentinel variants had opposite effect-directions (“platform-discordant”) (Extended Data Figure 3, **Figure S5**). Importantly, the majority of platform-discordant credible sets (15/19) contained a missense variant for the encoded protein (Figure 2B; **Table S5; Supplemental Note 1**), suggesting that discordant genetic associations may be driven by platform differences in affinity probe binding.

Similar to prior studies ^12,17^, the 644 proteins with a *cis*-pQTL detected on both platforms had higher inter-platform correlation (mean Pearson’s r=0.58, SD=0.20) than proteins with no significant *cis*-pQTL (mean Pearson’s r=0.21, SD=0.30) (Figure 2C, **Table S6**). Furthermore, the correlation of proteins with a platform-concordant, significant *cis*-pQTL was generally higher (mean Pearson’s r = 0.61, SD=0.18) than the correlation of proteins with a platform-discordant *cis*-pQTL (mean Pearson’s r = 0.37, SD=0.20) or a *cis*-pQTL on one platform only (mean Pearson’s r = 0.23, SD=0.28) (Figure 2B, 2C, **Table S6**).

As PAVs that drive putative epitope effects may differ in frequency between ancestry groups, PAVs may be one driver of inter-ancestry heterogeneity in cross-platform correlation (Figure 1). Overall, 72 proteins on SomaScan and 84 proteins on Olink were associated with *cis*-pQTL containing highly ancestry-differentiated PAVs (Chi-Square > 202, the 75th X^2^ percentile among variants tested in MESA). Indeed, we observed a higher rate of ancestry-differentiated *cis*-pQTL containing PAVs among the 80 proteins with Bonferroni-significant heterogeneity in inter-platform correlation estimates between ancestries (Cochran’s Q P<0.05/2,708) (16% compared to 5.5% (118/2,157) of proteins overall) (**Table 1**).

### Orthogonal genetic evidence to characterize *cis*-pQTL

Incorporating orthogonal lines of genetic evidence, such as expression quantitative trait loci (eQTL), or genetic variant associations with transcript abundance, may increase confidence that pQTL associations capture true alterations in protein abundance. Consistent with prior reports ^17^,^19^, we found that platform-concordant *cis*-pQTL signals were most likely to contain a GTEx v8 eQTL sentinel variant for the protein encoding gene in the credible set, compared to platform-specific, or platform-discordant signals. Thirty-six percent of platform-concordant pQTL credible set pairs, for 207 unique proteins, contained a GTEx eQTL with a concordant direction of effect, compared to 18% and 22% of SomaScan and Olink “specific” *cis*-pQTL, respectively (**Table S7**). Among the 15 platform-discordant, PAV-driven *cis-*pQTL, 4 had a reported genetic effect on transcript abundance in one or more tissues; the eQTL direction of effect agreed with the SomaScan pQTL direction of effect for 2 proteins (CTSS, HDGF) and with Olink *cis*-pQTL effect-direction for 2 proteins (HNMT, ACP1). Concordant eQTL evidence may support that pQTL associations detected on one platform only capture true differences in abundance. 9% (13/139) of SomaScan-specific PAV-containing pQTL credible sets also contain a concordant GTEx *cis*-eQTL, compared to 6% (14/208) of Olink-specific PAV credible sets (**Table S7**). The majority of *cis*-pQTL credible sets identified on each platform contained a GWAS catalog sentinel variant (**Table 2, Table ST3, Table ST4**).

While mass-spectrometry (MS)-based pQTL studies are relatively limited in size and number, comparing affinity-based pQTL to pQTL identified in MS studies may facilitate signal interpretation. A small proportion of affinity-identified pQTL (0.4% SomaScan, 0.6% Olink) harbor a previously reported MS-pQTL sentinel variant in the credible set (**Table 2; Table S7, ST3, ST4**). MS-pQTL studies may also help differentiate PAV-containing pQTL that do not reflect differences in overall protein abundance, but may, for example, reflect differences in specific peptide abundances, altered affinity reagent binding, or technical errors. Such “MS-PAV” variants hypothesized to drive epitope effects for affinity-based platforms accounted for a subset of PAV-containing signals, including platform-discordant signals for 2 proteins, APOL1 and PGLYRP2 (**Table S7, ST3, ST4**). Hence, MS pQTL findings may be used to better disentangle true biological abundance signals from artefactual signals.

### *trans*-pQTL identification and pleiotropy

**Extended Data Figure 4.**
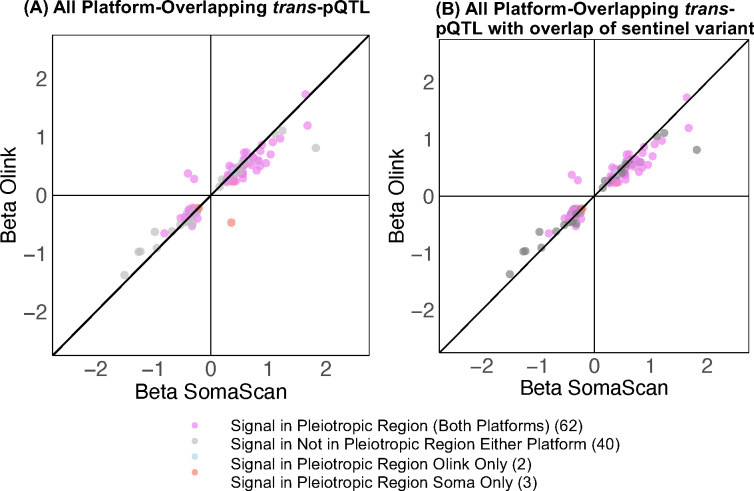
Sentinel variant *trans-*pQTL effect-size comparison for overlapping *trans*-pQTL signals (defined by credible sets) identified in analysis of one probe per platform, per protein, with points colored according to whether a credible set was located in a pleiotropic region on both platfoms, SomaScan only, Olink only, or neither platorm. (A) Effect-size comparison across all platform-overlapping credible sets. (B) Effect-size comparison across platform-overlapping credible sets for which at least one platform’s sentinel variant shared.

Associations between protein levels and genetic variants that are not in the vicinity of the protein encoding gene are also commonly detected in large scale studies and may reflect a host of biological or technical factors. Upon mapping *trans*-pQTL (pQTL >1 Mb of protein-encoding gene TSS) for SomaScan and Olink protein measures, we identified 424 significant *trans*-pQTL credible sets (credible set contains at least one variant with p<1×10^−11^) for 529 distinct proteins on SomaScan, and 315 for 262 proteins on Olink (Figure 2A & D, **Table 2, Table S8, S9, S10**, Extended Data Figure 4). While proteins with a *trans*-pQTL on both platforms exhibited higher inter-platform correlation (N=129, mean Pearson’s r=0.55, SD=0.26) (Figure 2D,E), proteins with a significant *trans*-pQTL on one platform only generally exhibited lower inter-platform correlation estimates (N=428, mean Pearson’s r=0.21, SD=0.29) than proteins with no *trans*-pQTL at all (N=1600, mean Pearson’s r=0.34, SD=0.31) (**Table S10**).

*trans*-pQTL associated with several protein measures may, among many other potential mechanisms, reflect non-specific affinity-probe binding or alterations to proteins involved in depositing PTMs that may interfere with affinity-probe binding. We identified 10 pleiotropic *trans*-pQTL regions that were associated with at least 5 proteins on both platforms, including well-characterized *ABO, FUT2,* and *F12*. Nine *trans*-pQTL regions were pleiotropic for SomaScan protein measures only (significant *trans*-pQTL associations with >=5 proteins on SomaScan, 0 or 1 on Olink), including *APOE, F5,* and *HPX*. The *FUT6; FUT3* region was identified as pleiotropic for Olink protein measures only in the main analysis (**Table S11, Table S12**). While proteins associated with a pleiotropic region on both platforms had highly correlated measures (N=67, mean Pearson’s r=0.57, SD=0.23), the 136 proteins associated with a *trans*-pQTL region pleiotropic for SomaScan only exhibited low inter-platform correlation (mean Pearson’s r=0.02, SD=0.21).

### Impact of limit of detection on protein measure correlation and pQTL detection

Limit of detection (LOD) refers to the lowest quantity of protein that can reliably be detected by an assay. The LOD for an assay may impact protein quantification precision or pQTL detection, particularly for low-abundance proteins. Here, the majority (2,155/2,157) of SomaScan proteins had >50% of measures above the company-reported LOD, while 63% (1,361/2,157) of Olink proteins measured had >50% of measures above the LOD (calculated per company-recommendations) (**Figure S6**; **Table S13**). While proteins with >50% measures above LOD were modestly correlated (N=1,361, median Pearson’s r=0.43, SD=0.32), proteins with <50% measures above LOD exhibited low inter-platform correlation (N=796, median Pearson’s r=0.04, SD=0.32), consistent with prior reports (**Table S14; Table ST15**) ^23^. 83% and 85% of proteins with a *cis*-pQTL or *trans*-pQTL detected on Olink had at least 50% of measures above the LOD, respectively. This suggests that additional scrutiny of how to best handle below LOD measures may be necessary for pQTL and other proteomics analyses, especially in cross-platform studies.

### Epidemiological protein associations

**Extended Data Figure 5.**
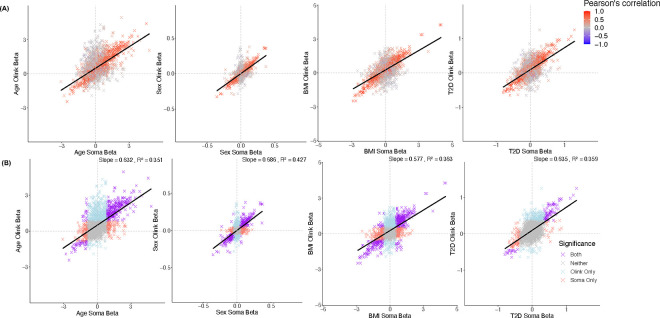
Effect size comparison of SomaScan and Olink phenotype~protein associations, for age, sex, body mass index (BMI), and type 2 diabetes (T2D) colored by (A) inter-platform correlation and (B) Bonferroni (p<0.05/2,708) significance of association on both platforms (purple), SomaScan only (red), Olink only (blue), or neither platform.

Proteomics measurements are increasingly used to understand how proteins influence traits and diseases; however, inaccurate protein measures may confound observations. Here, similar proportions of SomaScan and Olink protein measures were associated with age, sex, BMI, and type 2 diabetes (T2D) case/control status at Bonferroni significance threshold (**Table S16; Table S17**). Across all proteins, effect sizes for each phenotype were modestly correlated (Pearson’s r ~0.6) (Extended Data Figure 5). Among each phenotype, proteins with a Bonferroni significant phenotype association on both platforms were more likely to associate with a *cis*-pQTL on both platforms (**Table S16**). For age, sex, and BMI, we observed a small number of 6 to 25 proteins with platform-discordant, Bonferroni significant associations on both platforms (**Table S16**), suggesting that differences in protein measurements may indeed result in differences in epidemiological associations.

### Adjustment for ancestry-differentiated, platform-discordant pQTL improves concordance of genetic and epidemiological associations

**Extended Data Figure 6.**
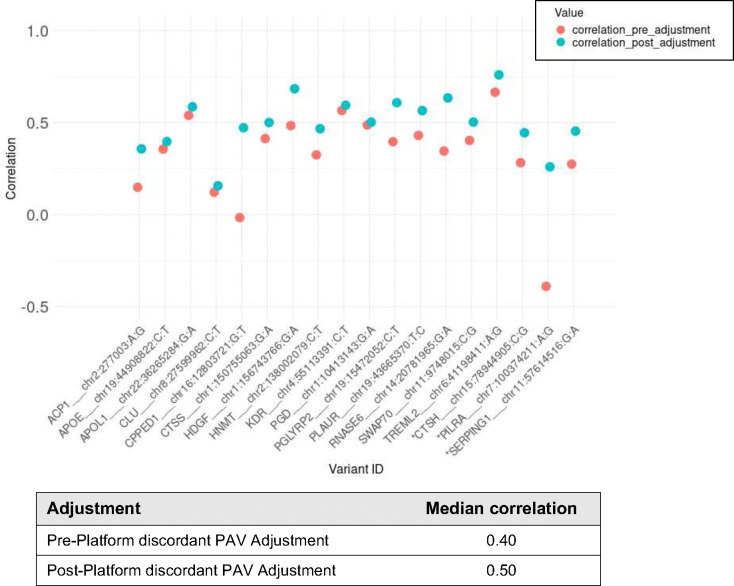
Inter-platform correlation pre- and post-adjustment for platform-discordant PAV. 15/18 proteins with a platform-discordant PAV correspond to probes included in main analysis. Protein/PAV pairs marked with asterisk correspond to probes not included in main analysis.

If the platform discordance for a given protein measure is largely driven by genetic variation, we hypothesized that adjustment for PAVs putatively driving inter-platform variation may improve concordance of protein measures and strengthen downstream epidemiological and genetic associations not reflecting assay interference. For the proteins associated with a platform-discordant *cis*-pQTL PAV, we adjusted each platform’s measures for copies of the PAV and computed inter-platform correlation estimates with residual measures. Prior to PAV-adjustment, the mean inter-platform correlation estimate for the 15 proteins was 0.38 (SD=0.18), with estimates ranging from −0.015 to 0.67. Post PAV-adjustment, the mean inter-platform correlation increased to 0.51 (SD=0.15), with estimates ranging from 0.15 to 0.76 (results for all probes in **Table S5**). Inter-platform correlation improved for all these proteins (mean improvement=0.14) (Extended Data Figure 6).

**Extended Data Figure 7.**
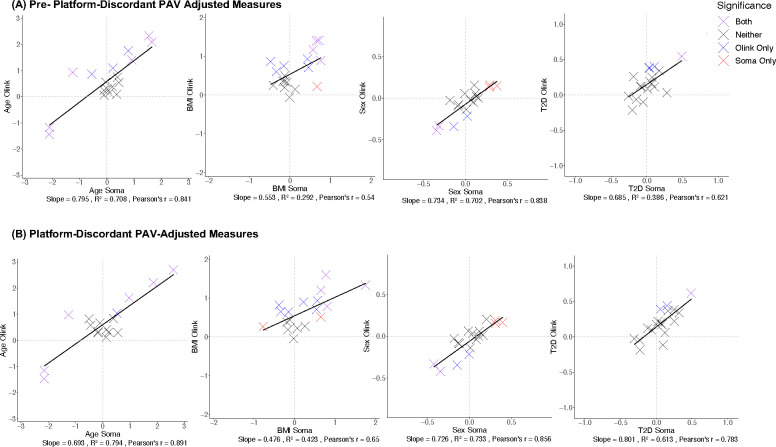
Effect-sizes from phenotypic regressions of protein abundance with age, sex, body mass index (BMI), and type 2 diabetes (T2D) for the 18 proteins with a platform-discordant PAV association in the full analysis of all probe pairs per overlapping protein target (A) Pre-PAV adjustment and (B) Post-PAV adjustment with points colored according to Bonferroni significance (p<0.05/2,708) of protein-phenotype association on both platforms (purple), SomaScan only (red), Olink only (blue), or neither platform.

**Extended Data Figure 8.**
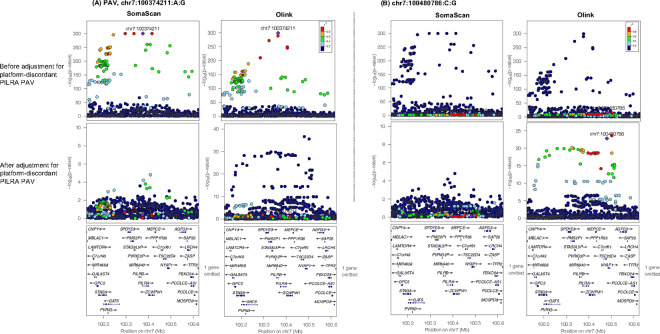
Locus Zoom plot displaying the 250KB window around the platform-discordant PILRA PAV before (top) and after (bottom) adjustment for the platform-discordant PAV (chr7:100374211:A:G) on SomaScan vs. Olink, with r^2^ values derived in MESA study participants. (A, Top) Platform-discordant PAV signal before adjustment on SomaScan and Olink (A, Bottom) Platform-discordant PAV signal after adjustment. As expected, this signal is attenuated to non-significance following adjustment. (B, Top) Prior to adjustment for the PAV, a second, non-coding signal in the region, led by chr7:100480786:C:G, is significantly associated with Olink PILRA measures. (B, Bottom) Following adjustment for the platform-discordant PAV, this signal becomes more significant on Olink. The Y-axes differ in scale between plots.

**Extended Data Figure 9.**
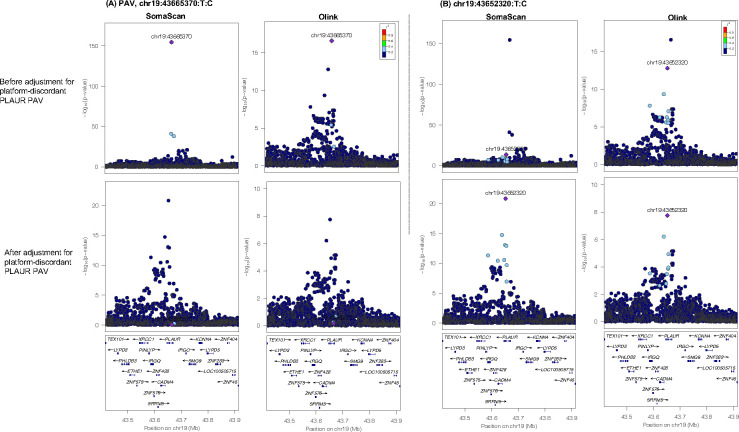
Locus Zoom plot displaying the 250KB window around the platform-discordant PLAUR PAV pre- (top) and post-(bottom) adjustment for the platform-discordant PAV (chr19:43665370:T:C) on SomaScan vs. Olink, with r^2^ values derived in MESA study participants. (A, Top) Platform-discordant PAV signal before adjustment on SomaScan and Olink (A, Bottom) Platform-discordant PAV signal after adjustment. As expected, this signal is attenuated to non-significance following adjustment. (B, Top) Prior to adjustment for the PAV, a second, non-coding signal in the region, led by chr19:43652320:T:C, is significantly associated with Olink PILRA measures. (B, Bottom) Following adjustment for the platform-discordant PAV, this signal becomes more significant on Olink. The Y-axes differ in scale between plots.

Next, we evaluated the impact of PAV adjustment on protein-phenotype associations by regressing adjusted measures with age, sex, BMI, and T2D case-control status. For proteins with a platform-discordant PAV, we observed an improved correlation of effect sizes observed among protein-phenotype associations when using PAV-adjusted measures as input (**Table S5**, Extended Data Figure 7). Additionally, when we performed *cis*-pQTL mapping, adjusting for PAV copies (**Tables S18, S19**), a subset of significant *cis*-pQTL associations were strengthened (8 SomaScan, 10 Olink) (**Tables S20, S21**), and 5 and 2 *cis*-pQTL associations on SomaScan and Olink, respectively, became significant (**Table S22**) (Extended Data Figure 8, 9, **Supplemental Note 3**), demonstrating that adjustment for putative assay interference effects may strengthen potential for biological discovery.

**Figure 3.**
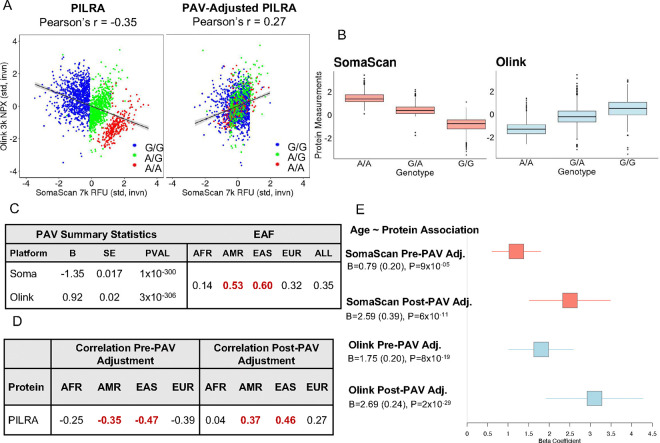
Adjustment for PILRA-associated PAV improves inter-platform correlation of measures and strengthens association with age. (A) Olink vs. SomaScan standardized and inverse normal transformed normalized protein expression (NPX) and relative fluorescence units (RFU), respectively, pre adjustment for PAV rs1859788 (chr7:100374211:A:G; p.Arg78Gly) (left) and post-PAV adjustment (right). (B) SomaScan and Olink PILRA measures by genotype. (C) pQTL summary statistics and effect allele frequencies per MESA ancestry-group. Red EAF values denote ancestries among which variant is most common (here, AMR and EAS). (D) Inter-platform protein measure correlation per ancestry pre- and post-PAV adjustment. Values highlighted in red indicate which ancestry groups experienced the largest improvement in correlation post-PAV adjustment. (E) Forest plot of age association statistics for SomaScan and Olink pre- and post-PAV adjustment.

One protein with platform-discordant measures that exemplifies the impact of PAV adjustment is paired immunoglobulin-like type 2 receptor alpha (PILRA). This protein showed strong platform-discordant measures (Pearson’s r = −0.35) across the present and prior studies ^16,17^ (Figure 3A). A platform-discordant *cis*-pQTL for PILRA is driven by an ancestry-differentiated missense-variant [rs1859788 G>A, PILRA, p.Gly78Arg, SomaScan: B=−1.34 (0.017), p=1×10^−300^, Olink: B=0.92 (0.020), p=2.98×10^−300^] most common among EAS (EAF=0.60) and AMR (EAF= 0.53) participants, compared to AFR (EAF=0.14) and EUR (EAF=0.32) participants (Figure 3B, 3C). Upon adjusting for copies of the PAV allele, the correlation of residual PILRA measures improved to 0.27 (Figure 3A). Inter-platform protein measure correlation improved most among AMR and EAS participants (Figure 3D, **S14**). Furthermore, incorporating PAV-adjusted PILRA measures into epidemiological models improved the strength and concordance of downstream PILRA-age associations (Figure 3E).

**Figure 4.**
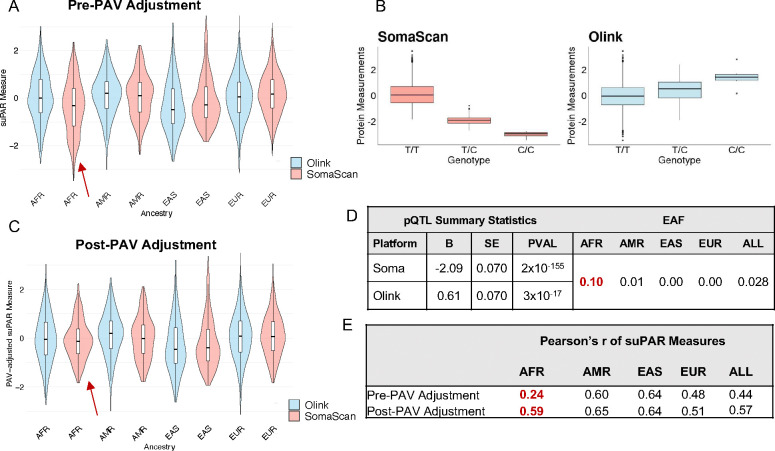
Adjustment for suPAR-associated, ancestry-differentiated PAV improves correlation of suPAR measures across all participants, with improvement driven by individuals of African ancestry. (A) suPAR measures per platform, per-ancestry, pre-adjustment for PAV rs399145 (chr19:43665370:T:C; p.Thr86Ala). (B) SomaScan and Olink suPAR measures by genotype. (C) suPAR measurements per-platform, per-ancestry, post-PAV adjustment. (D) suPAR pQTL summary statistics per platform and effect allele frequencies per ancestry group in MESA. Red EAF values denote ancestries among which variant is most common (here, AFR). (E) Inter-platform correlation of suPAR measures before and after PAV adjustment. Values highlighted in red indicate which ancestry groups experienced the largest improvement in correlation post-PAV adjustment (here, **AFR).**

Soluble urokinase plasminogen activator receptor (suPAR) is another protein associated with an ancestry-differentiated, platform-discordant PAV. suPAR measures are modestly correlated across all participants (Pearson’s r=0.43), but there is significant heterogeneity in inter-platform correlation estimates between ancestries (Cochran’s Q = 33.5, Cochran’s Q p=1.65×10^−12^), with lower correlation estimates among individuals clustering with the AFR ancestry reference population (Pearson’s r = 0.24, Figure 4A, 4D, 4E). Additionally, a platform-discordant *cis*-pQTL driven by a missense variant [rs399145 T>C, PLAUR, p.Thr86Ala SomaScan: B=−2.09 (0.070), p=2.03×10–155, Olink: B=0.61 (0.070), p=2.58×10–17] common among individuals with African ancestry [EAF in MESA participants AFR=0.10, AMR = 0.009, EAS =0.000, EUR =0.004, EAF X^2^=230.7 (ancestry-differentiated), X^2^ p=9.49×10^−50^] (Figure 4B) associates with suPAR measures. Upon adjusting for copies of the PAV allele, the correlation of protein measures improved from 0.44 to 0.57, with the largest improvement in inter-platform correlation estimates among individuals of African ancestry (Pearson’s r = 0.59 post-PAV adjustment) (Figure 4C). In summary, adjustment for PAVs may improve concordance of protein measures and downstream associations. Additional examples for APOL1 and CPPED1 proteins are in **Supplemental Note 4**.

## Discussion

As affinity-based proteomic platforms shape our understanding of genetics and disease, it is imperative to consider protein measure validity, and account for putative genetic drivers of affinity-based protein measure discordance across diverse populations. This study represents the first comprehensive comparison of affinity-based protein measure agreement and genetic signals in an ancestrally diverse cohort. We highlight proteins with systematic differences in assay measurements by ancestry and demonstrate that genetic variants with different frequencies across ancestries may contribute to these measurement differences. Moreover, we demonstrate that adjusting for these likely assay-interference variants can strengthen potential for biological discovery.

Among the 2,157 proteins with SomaScan 7k and Olink Explore 3072 measures compared here, we observed similar proportions of measures associated with *cis*-pQTL, and PAV-driven *cis*-pQTL, on each platform, consistent with single-ancestry genetic comparisons of earlier versions of SomaScan and Olink platforms ^12,16,17^. Proteins associated with concordant *cis-* genetic signals exhibited the highest average inter-platform correlation, increasing confidence that the affinity probes for these proteins captured the same entities and may have high potential for cross-platform meta-analysis.

However, we also demonstrated instances of coding *cis*-pQTL genetic variation (PAVs)–including highly ancestry-differentiated variation – that may impact the target protein structure or affinity reagent binding. For protein measures associated with PAVs on both platforms with differing directions of effect, adjustment for the PAV improved protein measure correlation and resulted in improved strength and concordance of some genetic and epidemiological signals, even allowing detection of new significant associations. Hence, PAV-adjustment may provide an avenue of accounting for potential epitope-binding effects in studies with data from one platform only. Although only 19 proteins associated with a platform-discordant PAV, several are considered potential biomarkers or drug targets. For example, suPAR is a potential biomarker or therapeutic target for kidney inflammation ^24,25^. As sample sizes and power for pQTL detection and platform protein coverage expand, we predict more such proteins will be identified, underscoring the importance of considering differential epitope binding effects in proteomic analyses.

More than 322 platform-specific *cis*-PAVs were identified; more work is needed to distinguish which of these QTLs are due to alterations in affinity reagent binding versus protein abundance. Integration of larger MS-pQTL datasets and eQTL datasets will facilitate identification of epitope-binding artifact pQTLs and help differentiate, in situations of discordant pQTL effects, which platform better reflects true protein abundance.

Interpretation of affinity-based *trans*-pQTL, particularly pleiotropic *trans*-pQTL, is challenging. While detection of any platform-shared *trans*-pQTL association for a protein corresponds with high inter-platform correlation, proteins associated with regions pleiotropic on SomaScan only had low inter-platform correlation. Existing affinity-based proteomics studies exploring *trans*-pQTL often interpret pleiotropic *trans*-pQTL as biologically relevant signals which may help confirm appropriate assay performance ^26,27^. While pleiotropic regulators certainly exist and are relevant in metabolism and disease risk ^28^, it is challenging to interpret whether pleiotropic *trans*-pQTL reflect true differences in protein abundances, systematic alterations to PTMs that interact with epitope binding, or other factors that may influence interactions, especially in the absence of cross-platform replication or orthogonal validation with a different assay.

Proteomics platform measurements for a protein may vary across diverse ancestries. Here, 80 proteins had significantly heterogeneous correlation across ancestries. These proteins were enriched for association with PAVs with ancestry-differentiated allele frequencies, supporting epitope-binding effects as one driver of cross-ancestry differences in platform agreement. Adjustment for platform-discordant, ancestry-differentiated PAVs generally reduced cross-ancestry heterogeneity in inter-platform correlation estimates. However, the improvement in inter-platform correlation across ancestry groups was not linear, suggesting the interplay of environmental or other undetected genetic effects. Confounding by differential recruitment of participants of varied ancestry by site, or other environmental or clinical factors, may also contribute to ancestry-heterogeneity in correlation estimates.

Failure to account for assay-interfering PAVs in studies of circulating proteins may hinder detection of disease-relevant associations or putative biomarkers. These PAVs may further bias Mendelian Randomization (MR) results if used as genetic instruments for circulating protein abundances ^29^. Similarly, if proteomics platforms are employed clinically, failure to account for genetic variation that confounds protein abundance measures may result in systematically inaccurate measures among carriers of such genetic variants, thereby confounding diagnosis/prognosis.

### Limitations

While this study is currently the most diverse platform-comparison, representation of non-European ancestry groups remains limited. Additional studies with increased numbers of non-European ancestry participants will facilitate identification of genetic drivers of ancestry-heterogeneity. Due to sample size constraints, ancestry clusters are also derived at the continental level in this study; future studies should consider heterogeneity within continents for cross-platform protein correlation and protein measurement impacting pQTLs.

Additionally, this analysis is limited to the 2,157 protein targets measured on both platforms. However, SomaScan 7k and Olink Explore 3072 investigated in this study measure up to 7,000 and 3,072 proteins, respectively. Furthermore, each platform has released a new version, with SomaScan and Olink Explore now measuring up to 11,000 and 5,400 proteins, respectively. We cannot assume that the same patterns observed in this study hold true in newer versions of the platforms, particularly as platforms expand into coverage of lower abundance proteins and more intracellular proteins. These analyses should be repeated as data on large numbers of overlapping samples from ancestrally diverse participants accrues on the most recent SomaScan 11k and Olink 5k platforms. Furthermore, power for pQTL (particularly *trans*-pQTL) discovery is limited by sample size. Additional evaluation of pQTL across increased sample sizes will likely yield additional associations.

Here, and in prior comparisons, the inter-platform correlation of protein measures serves as a proxy for on-target epitope binding. While consistent rank-based protein measures increase confidence in protein measures, it remains possible that neither platform provides accurate protein measures. It is especially challenging to interpret platform-discordant measures, though metrics like *cis*-pQTL presence may provide clues. Further platform comparisons incorporating MS protein measures are needed. The release of additional information regarding affinity-probe confirmation and target domains from SomaLogic and Olink would prove useful for research interpretation of affinity probe binding.

## Methods

### Study sample

The Multi-Ethnic Study of Atherosclerosis (MESA) is a prospective, population-based cohort study of 6,814 men and women, aged 45 to 84 years, with recruitment from 6 US clinical centers beginning in 2000 ^30^. At the baseline exam, 53% of participants self-identified as female, 38% as non-Hispanic White, 28% as African American, 12% as Chinese, and 22% as “Spanish, Hispanic, or Latino” (subjects with both Caribbean and Mexican/Central American ancestry are represented). All participants provided written informed consent, including for genetic study. MESA has been approved by the institutional review boards of each field center, the data coordinating center, and the genetic analysis center. The Institutional Review Board at the Lundquist Institute for Biomedical Innovation also reviewed and approved this project.

### Blood sample collection

The MESA exam 1 protocol for blood sample collection and processing has been previously described ^30,31^. In brief, extracted blood was added to EDTA and mixed for at least 30 seconds. Blood-EDTA samples were stored upright on ice for no longer than 30 minutes following extraction. Centrifugation was performed at 2000g for 15 minutes, or 3000g for 10 minutes. Centrifuged samples were placed on ice; plasma was extracted into 0.5mL aliquots. Cryovials were frozen and stored in upright position at −70°C. Samples were shipped on dry ice. Proteins in plasma samples collected in this manner have been shown to be stable over time ^32^.

### Genetic ancestry assignments via clustering

Continental ancestry of each subject was assigned using Admixture ^33–35^ in a supervised analysis with reference genotypes from the 1000 Genomes project ^36^ and the Human Genetic Diversity Panel (HGDP) ^37^. Uniformly called genotype data for MESA, 1000 Genomes, and HGDP were obtained from TOPMed whole genome sequence data ^38^. MESA subjects were assigned to a 1000G/HGDP sub-population based on similarity greater than 50% to reference (with a small number of MESA participants (n=44) unclassified using this threshold).

### Proteomics measures

Plasma samples for a subset of participants from the baseline MESA exam 1 (2000–2002) were assayed on both SomaScan 7k and Olink Explore 3072. Measures from both platforms were obtained in one batch. Probes were matched to UniProt IDs using reference files from SomaLogic and Olink, respectively. Here, we filtered protein measures to those obtained for human protein targets. RFU and NPX measures obtained from each platform were standardized and rank-based inverse normal transformed prior to conducting analyses.

### Protein measure comparison and inter-ancestry heterogeneity of protein measures

Pearson’s correlation coefficients were computed for transformed SomaScan measures against Olink measures, as well as for ANML-SomaScan measures against Olink measures, using data from all 1,930 contributing participants. We also computed Pearson’s correlation coefficients among individuals stratified by ancestry group, excluding the 44 participants who could not be assigned to ancestry groups using the criteria defined. To evaluate whether correlation estimates for a given protein differed significantly across ancestry groups, we computed the Fisher’s R-to-Z transformation on per-ancestry Pearson’s r estimates and performed the Cochran’s Q test of heterogeneity across resulting Z-scores.

### Whole Genome Sequencing

TOPMed WGS methods were described previously ^38^. Briefly, WGS was conducted at six sequencing centers (mean depth >30X, Illumina HiSeq X Ten instruments). Joint variant discovery and genotype calling were conducted by the TOPMed Informatics Research Center (IRC) across all TOPMed studies using the GotCloud pipeline, resulting in a single genotype call set encompassing all of TOPMed (TOPMed Freeze 10). Variant quality control was also performed centrally by the TOPMed IRC. Sample quality control was performed by the TOPMed Data Coordinating Center (DCC).

### pQTL mapping

Prior to QTL analyses, inverse-normal transformed protein measures from each platform were residualized based on participant age, sex, MESA recruitment site, protein measure plate number, 10 genotype PCs, and 10 protein PCs. Notably, we performed QTL mapping with ANML-SomaScan measures. Gencode version 45 gene coordinates were used to define TSS ^39^. If gene coordinates could not be found in Gencode, Ref Seq gene coordinates downloaded via the USCS Genome Browser on June 5, 2023 were used. *Cis*- and *trans*-QTL mapping was performed within tensorQTL ^40^, adjusting for the same set of covariates as listed above. Here, *cis*-pQTL is defined as pQTL within 1Mb of the TSS, and *trans*-pQTL is defined as >1Mb from the TSS. Fine-mapping of resulting *cis*- and *trans*- signals was performed using the cis.susie and trans.susie functions in tensorQTL, respectively, to obtain credible sets of variants within each signal for each platform. Significant credible sets were defined as *cis*-pQTL credible sets containing at least one variant associated with a protein with p<5×10^−08^, or *trans*-pQTL credible sets containing at least one variant associated with a protein with p<1×10^−11^. After observing a small subset of cases for which SuSiE output credible sets containing variants in high LD, we filtered credible sets for the same trait that contain variants in LD (r^2^>0.1) across participants to retain only the credible set with the more significant lead variant.

### Identification of ancestry-differentiated variants

To identify variants with differing EAF by ancestry, we obtained EAF of each variant within each contributing ancestry group in MESA using plink version 3 ^41^ (www.pngu.mgh.harvard.edu/purcell/plink/), specifying the TOPMed reference allele as the reference allele for each ancestry. We then computed Chi-Square test statistics and p-values across EAF values for each variant. Given the large number (10,272,095/12,273,390) of highly significant variants, even following multiple-testing correction (p<0.05/12,273,390), we consider a Chi-Square value in the 75th percentile across all MESA participants (X^2^>0.75) as evidence of ancestry-differentiation.

### Cross-platform pQTL signal comparison

Here, we considered pQTL credible sets for a given protein “platform-overlapping” if there was at least one shared variant across the credible sets associated with the same protein measure on each platform. If neither platform’s signal contained the sentinel variant for the other platform, we did not compare the concordance of effects. Genetic signals that did overlap for at least one platform’s sentinel variant were categorized as platform-concordant or platform-discordant based on direction of association of the lead variant with each platform’s protein abundance measure (**Figure S4**). Platform-concordant signals are significant pQTL signals with concordant directions of association with the given protein on both platforms, whereas platform-discordant signals are significant pQTL signals with differing directions of effect with the given protein across platforms.

### Assessment of protein subcellular location

Human Protein Atlas ^42,43^ was used to ascribe protein subcellular locations as membrane, secreted, or intracellular as previously described ^4,17^. Briefly, proteins were considered membrane proteins if annotated by HPA only as membrane proteins, secreted proteins if annotated only as secreted proteins, and intracellular if annotated by HPA as both or neither.

### Identifying protein altering variants

Functional annotation and further characterization of variants was performed via WGSA 0.95 ^44^, which integrates variant annotations from Variant Effect Predictor (VEP) ^45^, Annovar ^46^, and SNPEff ^47^. Here, we used report predicted consequences output by VEP ^45^, annotated relative to Ensembl, in the main analyses. Protein-altering variants were defined as VEP-Ensembl annotated missense variants, start-loss, stop-gain, or stop-loss variants. The vast majority of PAVs were missense variants. For *cis*- and *trans*-pQTL main analyses, we report numbers of signals reported by VEP to alter the protein-encoding gene.

### Affinity pQTL overlap with GTEx *cis-e*QTL

To assess whether pQTL may exhibit shared effects with GTEx expression quantitative trait loci (eQTL), we evaluated whether any variants within identified *cis*- and *trans*-pQTL credible sets are reported lead-eQTL (for either primary or secondary signals, based on conditional analysis) in GTEx version 8 ^48^. When comparing overlap between *cis*-pQTL signals and GTEx eQTL signals, we filtered GTEx eQTL counts by whether the observed eQTL corresponded to the same protein-encoding gene. Notably, the GTEx consortium reports eQTL for several tissues. To label a pQTL direction of effect as concordant with the GTEx reported eQTL direction of effect, we required the pQTL and GTEx eQTL direction of effect for all tissues in which the eQTL was significant. A small subset of pQTL signals (28 credible sets on SomaScan, 27 credible sets on Olink) contained more than one distinct eQTL signal, with conflicting directions of effect. We excluded these signals from effect-direction comparisons.

### Affinity pQTL overlap with MS-pQTL

To assess whether pQTL regions are previously reported MS-pQTL, we evaluated whether any sentinel SEER MS-pQTL in either the 320 Qatar Metabolomics Study of Diabetes (QMDiab) ^10^ or the larger the Tarkin Study ^10^ of 1,980 participants were present in our SomaScan or Olink pQTL credible sets, and determined whether these signals were associated with the same protein by comparing associated UniProt IDs. We further assessed overlap of affinity-based pQTL with MS-PAVs, which are MS-based variants hypothesized by Suhre et. al ^10^ to reflect impacts on protein quantification by PAV, and not true differences in protein abundance.

### MESA pQTL overlap with deCODE and UK BioBank reported pQTL

To assess whether pQTL regions identified in the present study were previously reported in the deCODE-based SomaScan version 4 pQTL study (n=35,559 Icelanders) ^1^ or the UK Biobank Olink Explore 3072 pQTL study (n=54,219 UKB Pharma-Proteomics-Project participants) ^5^, we evaluated whether the 1MB region surrounding the sentinel variant contained a previously reported sentinel variant for the same protein association from one of these two studies.

### MESA pQTL overlap with GWAS catalog variants

To evaluate whether pQTL have been previously associated with phenotypes, we assessed whether pQTL signals contained sentinel variant-trait associations reported in the GWAS Catalog (downloaded August 12, 2024) ^49^.

### Characterizing pleiotropic *trans*-pQTL regions

Several regions contained multiple *trans*-pQTL signals that often associated with one or more of the same proteins within and across platforms. Hence, we took a region-based, rather than a signal-based, approach to define platform-shared pleiotropy vs. “platform-differentiated” pleiotropy. For each platform, we defined *trans*-pQTL regions by finding credible sets with the most significant sentinel variant and defining a 1Mb window around these variants. If 1Mb windows overlapped, we merged into one. We subsequently compared regions identified for each platform: if regions overlapped across platforms, we defined them as platform-shared.

Platform-shared pleiotropic regions were defined as overlapping *trans*-pQTL regions associated with at least 5 proteins on both platforms. Platform-specific pleiotropic regions were defined as regions associated with at least 5 proteins on one platform, and 0 or 1 protein on the other.

### Limit of detection

SomaScan global limit of detection (LOD) values were obtained from SomaLogic. Per company recommendations, we compared protein LOD values to ANML-normalized sample protein RFU measures. Olink LOD values were calculated from negative control data, per company recommendations. 186 negative controls samples from the 102 plates used in the present analysis were used for calculation of LOD. Protein measures were filtered to intensity normalized values (excluding amplification, incubation, and extension controls). LOD values were calculated for each protein across all plates by taking the median of the negative control measure + 3 fixed standard deviations. We compared LOD values to non-transformed Olink sample NPX values.

### Epidemiological analyses

Proteins were associated with participant age and BMI using linear regression models in R ^50^ adjusted for plate number, and site in age and sex models, and additionally adjusted for age and sex in BMI models. Proteins were also associated with sex and prevalent T2D using logistic regression, with adjustment for age and site (as well as sex in T2D model).

### Evaluation of genetic variant impacts on concordance of protein measures and epidemiological associations

To evaluate the impact of *cis*-protein altering genetic variation on protein abundances, we obtained counts of PAV alleles present in each participant and adjusted protein measures for copies of the effect allele in these variants. Protein residuals from these models were used to evaluate the concordance of adjusted-protein measures.

Furthermore, these protein residuals were used as input in epidemiological analyses and *cis*-pQTL mapping to explore the impact of PAV adjustment on downstream associations. To determine cases where adjustment for PAV resulted in more concordant phenotypic associations, we performed effect-size heterogeneity tests across SomaScan and Olink results obtained using 1) non-PAV adjusted protein measures as model input, then 2) using PAV-adjusted protein measures as input and compared proteins with Bonferroni significant effect-size heterogeneity pre- vs. post-PAV adjustment.

To evaluate the impact of platform-specific pleiotropic *trans*-pQTL protein altering genetic variation on protein abundances, we obtained counts of sentinel *trans*-pQTL alleles in credible sets mapping to regions defined as platform-specific pleiotropic and adjusted protein measures for copies of the effect allele in these variants.

### Adjustment for protein altering genetic variants in QTL analyses

To determine the impacts of platform-discordant PAV adjustment on *cis*-pQTL, we repeated *cis*-pQTL mapping and fine-mapping analyses for the 19 proteins associated with platform-discordant PAVs using the previously described tensorQTL pipeline, this time adjusting for copies of the respective PAV as a covariate. We then determined whether *cis-*pQTL signal significance increased or decreased following PAV adjustment by overlapping pre- and post-PAV adjusted credible sets obtained within each platform and comparing summary statistics at the lead variants. To account for a subset of signals that appeared to increase in significance post-PAV adjustment due to residual LD with the adjusted-PAV, we filtered PAV-adjusted credible sets that contained variants in LD with non-PAV adjusted credible sets. Here, “newly-associated” *cis-*pQTL are defined as *cis*-pQTL credible sets obtained in PAV-adjusted models that do not overlap, and are not in LD (R^2^>0.1) with variants in credible sets from pre-PAV adjusted models.

## Extended Data
